# An Epidemiological and Etiological Analysis of 5026 Peripheral Nerve Lesions from a European Level I Trauma Center

**DOI:** 10.3390/jpm12101673

**Published:** 2022-10-08

**Authors:** Martin Aman, Kim S. Zimmermann, Mirjam Thielen, Benjamin Thomas, Simeon Daeschler, Arne H. Boecker, Annette Stolle, Amir K. Bigdeli, Ulrich Kneser, Leila Harhaus

**Affiliations:** Department of Hand-, Plastic and Reconstructive Surgery, Burn Center, BG Trauma Center Ludwigshafen, Department of Hand-and Plastic Surgery, University of Heidelberg, 69117 Heidelberg, Germany

**Keywords:** peripheral nerve, nerve injury, trauma, epidemiology, nerve reconstruction, nerve treatment

## Abstract

Background: Peripheral nerve lesions are associated with debilitating long-term consequences. Albeit being essential for evidence-based clinical decision making, epidemiological and etiological data are scarce. We therefore aimed to comprehensively analyze epidemiological and etiological factors of peripheral nerve lesions in one of the largest cohorts. Methods: We screened a total of 110,667 patients treated at our level I trauma center between January 2012 and July 2020 for nerve lesions. Subsequently, demographics, etiologies, concomitant injuries, and lesion characteristics were analyzed. Results: A total of 5026 patients, predominantly young males suffering from non-work-related nerve injuries, were treated. Proximal levels of injury were more likely to be accompanied by fractures, whereas more distal injuries with concomitant vessel or tendon injury. Main causes were 54.6% lacerations. Acute traumatic nerve injury was treated within 24 h in 55.9% of cases. Conclusions: Given the young age of affected patients, early diagnosis and treatment in specialized centers may facilitate their early return to work and improve long-term functional outcomes. The data show the importance of a special attention on nerve injuries, which may be masked by large accompanying injuries. New findings on lesion characteristics of selected subgroups and accompanying circumstances can support a change in treatment strategies.

## 1. Introduction

Peripheral nerve lesions are devastating and often accompanied by prolonged regeneration and incomplete recovery. This leaves many patients with long-term sequelae, such as chronic pain or impaired motor or sensory function. The associated socioeconomic and psychosocial consequences are tremendous, with long sick leaves from work, potential occupational redeployment, and psychological strain, ultimately resulting in severe burdens for patients and healthcare providers as well as for society [1]. Due to the long regeneration times and the considerably short window for sufficient reinnervation of 18 months, an early and precise diagnosis and an individualized treatment of peripheral nerve lesions are crucial [2]. In cases of trauma, it is assumed that a high percentage of nerve injuries remain unidentified at the first clinical presentation, especially in closed injuries or injuries with multiple damaged structures [3].

Until now, there are only a few specialized centers who comprehensively manage patients with nerve lesions from the initial presentation onward, thus guaranteeing the right diagnostics, timing, and therapy for each individual case. Vast regions are undersupplied with such services [4,5]. The result is a high number of incompletely treated patients suffering from the severe long-term consequences as described above.

Therefore, transparently archiving and systematically presenting the epidemiological and etiological characteristics of nerve lesions with the help of a large database can help to sensitize centers seeing and treating patients with nerve lesions in order to improve the quality of treatment.

In this context, the currently available literature either provides clinical data deduced from small case series or consists of demographic data from large national insurance registries, but further details regarding mechanisms of injury or concomitant injuries are missing [6,7,8]. Moreover, differing demographics of countries regarding age, infrastructure, employment rates, and healthcare systems further limit their comparability in general.

Therefore, the objective of this study was to analyze the epidemiology in peripheral nerve lesions concerning demographics, cause of the lesion, involved nerves, level of injury and inpatient stay in a representative sample of patients treated for nerve lesions in a level I trauma center during a period of 8.5 years.

## 2. Materials and Methods

After obtaining approval by the local ethics committee (Mainz, Germany, reference number 2021-16091), all patients treated at our institution between January 2012 and July 2020 were screened retrospectively for cases of peripheral nerve lesions by using both the digital hospital information system and ICD-10 Classification.

### Exclusion and Inclusion Criteria

All patients with the presence of a peripheral nerve lesion were included. All cases in which the nerve lesion was only a secondary diagnosis and was not treated during the stay were excluded. Furthermore, indirect nerve lesions such as major or minor amputations or CRPS treatment were not included as they represent an individual ICD diagnosis.

Duration of inpatient treatment is displayed in “nights” in hospital to prevent interference in data analyses of outpatient and day-care patients.

Data acquisition was performed by two independent reviewers (M.A., K.S.Z.) in a pseudonymized manner. An anonymized database was then created using Microsoft Excel. After full data acquisition, SPSS Statistics Version 27 (IBM, Chicago, IL, USA) was used for statistical analysis. The data are reported as the mean of continuous data accompanied by the standard deviation and the mode (age, inpatient treatment, number of operations). Following the verification of normal distribution, differences in the distribution of continuous measures were assessed using the Student’s *t*-test (age, inpatient treatment), and the distribution of categorical factors was analyzed by means of the Pearson’s Chi-Square Test (weekly distribution, seasonal distribution). For variables with only two values, the test for binomial distribution was used to detect significant differences (sex, affected side). Statistical significance was determined with a two-sided *p*-value < 0.05 and an alpha level of 0.05 was chosen for all tests.

Data presentation was in accordance with SAMPL guidelines, and data are displayed as M (SD) = mean (standard deviation).

## 3. Results

### 3.1. Demographics

**Figure 1:**
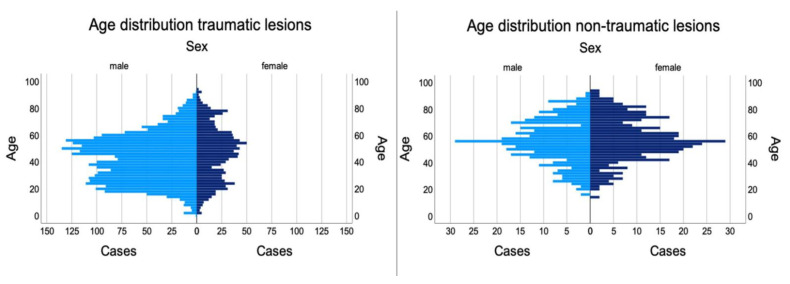
Age distribution of male and female patients suffering from peripheral nerve lesions.

We analyzed a total of 110,667 patients treated at our trauma center from January 2012 to July 2020. Thereof, 5026 cases with at least one peripheral nerve lesion were identified: 288 were children and adolescents under the age of 18 (5.7%), whereas 4738 were adults (94.3%). Mean patient age was 45.8 (18.6) years. Two peaks were found at the age of 24 and 56. Patients were predominantly male (3401; 67.7%) compared to 1625 (32.3%) female patients (*p* < 0.001) (Figure 1).

### 3.2. Traumatic vs. Non-Traumatic Lesions

Analyzing the mechanism of injury, we found 2921 acute traumatic injuries (52.2%). The other 47.8% (2679) had a non-traumatic or post traumatic cause such as compression (1401; 25%), neuroma formation (194; 3.5%), tumor (47; 0.8%), irritation (940; 16.8%), inflammation (11; 0.2%), and unclear cause (86; 1.5%).

Overall, intraoperative findings demonstrated intact continuity of nerves in 2608 cases (46.6%) despite preoperative loss of function. In contrast, 2506 cases (44.8%) had a full, and 415 (7.4%) an incomplete laceration of the nerve. Furthermore, 73.5 % of patients presenting with traumatic nerve injuries were male with a mean age of 43.2 (18.0) years. This varied significantly (*p* < 0.001) from non-traumatic patients, who were 52.3% female with a mean age of 56.2 (16.2) years.

Furthermore, 350 (6.8%) iatrogenic nerve lesions were observed in patients with a mean age of 51.8 (18.0) whereof 53.4% were female, not significantly varying from male patients (*p* > 0.05). Consider that inpatient treatment iatrogenic nerve lesions had a significantly longer stay (*p* < 0.05) of 9.2 (9.6) nights in hospital versus 7.6 (12.7) nights in traumatic injuries and 4.9 (13.6) nights of non-traumatic lesions. Hereby, the radial nerve was the leading nerve, being affected in 33.1% of the cases.

The majority of lesions was located at the upper extremity with 4439 (88.3%) cases. Furthermore, we found 494 (9.8%) nerve lesions in the lower extremity, 75 (1.5%) in the head and neck, and 18 (0.4%) in trunk or combined head/extremity lesions. In addition, 2363 lesions (47%) occurred on the right side, whereas 2614 (52%) were on the left side. This difference was statistically significant (*p* < 0.001). Only 48 cases (1%) had nerve lesions on both sides. In addition, 3517 (70%) were leisure accidents, whereas 1509 (30%) were work related injuries. Hereby 54.6% of lesions were caused by cuts, 22.2% had a non-traumatic cause such as compression, 9.8% falls, 7.0% motor vehicle injuries, 3.4% various trauma, 1.3% elongations, and 0.6% accounted for burns, gunshots, explosions, and bites each. Patients spent an average of 7.95 (13.04) nights in hospital, with a mode of two nights (799 cases, 17.2%) (Table 1).

In total, the most frequent lesions were injuries to the finger nerves (2089; 37.3%). Regarding lesioned main nerve trunks, 1255 median nerve (22.4%); 839 ulnar nerve (15%), 552 radial nerve (9.9%), and 250 peroneal nerve lesions (4.5%) were identified. In addition, 128 patients presented with brachial plexus injuries (2.3%), 90 with tibial nerve (1.6%), 76 with facial nerve (1.4%), 55 with sciatic nerve (1%), 38 with sural nerve (0.7%) and 29 (0.5%) with lateral cutaneous femoral nerve lesions (Table 2).

### 3.3. Lesion Level

**Figure 2:**
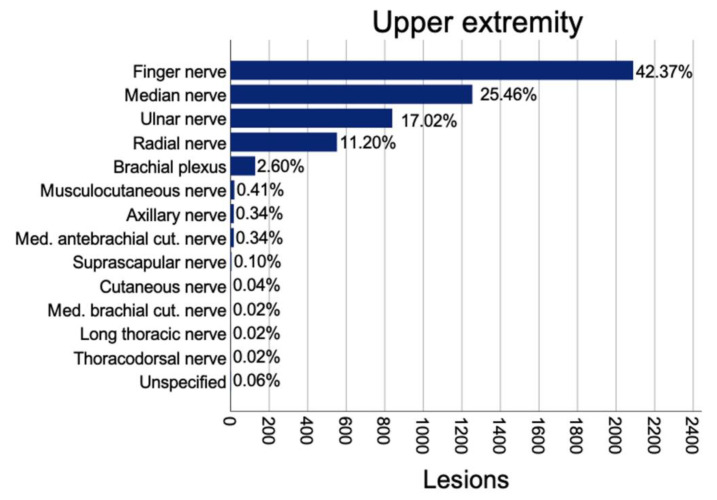
Prevalence of nerve lesions in the upper extremity.

Subgroup analyses based on the level of lesion were used to enhance the comparability of concomitant injuries and associated duration of inpatient treatment or number of follow up surgeries (Figure 2).

#### 3.3.1. Upper Extremity

At the level of the shoulder (n = 155), the most frequent lesions affected the brachial plexus (124; 80%), followed by isolated axillary (10; 6.5%) and radial nerve lesions (9; 5.8%). The main cause where motor vehicle accidents (70; 45.2 %), falls (34; 21.9%), and non-accidental causes such as compressions or tumors (25; 16.1%). Regarding concomitant injuries of tendons and muscles, nine cases had one (5.8%), two cases had two (1.3%) and 19 cases (12.3%) multiple (i.e., more than two) accompanying tendon or muscle lesions. Large blood vessel injuries comprised one (7; 4.5%), two (3; 1.9%), or multiple (18; 11.6%) affected blood vessels. In addition, 62 (40%) cases had a fracture at the level of the shoulder. Consequently, the mean duration of inpatient treatment amounted to 15.8 (17.6) nights with a mean of 1.3 (1.5) surgeries.

In this cohort, the nerve lesions were verified by intraoperative exploration and revealed elongation or irritation in 104 (67.1%) of cases. Twenty-three (14.8%) cases had unclear causes but intact nerves, 20 (12.9%) lacerations, thereof 17 cases with a full laceration of the nerve, three with an incomplete laceration, and four (2.6%) were due to a tumor. In addition, 84.6% of the traumatic injuries affected hereby the brachial plexus followed by 6.2% axillary nerve injuries.

At the level of the upper arm and elbow, a total of 635 nerve lesions were found. Most commonly affected was the ulnar nerve (347; 54.6%) followed by the radial nerve (207; 32.6%), median nerve (56; 8.8%), and musculocutaneus nerve (10; 1.6%). Concomitant injuries were 51 (8%) blood vessel lacerations (at least one affected vessel) and 67 (10.6%) muscle injuries. Fractures of the humerus were found in 199 (31.3%) cases. Patients spent a mean of 9.3 (13.9) nights in hospital with a mean of 1.24 (0.98) operations. Most common causes were non-traumatic (218; 34.3%), followed by falls (192; 30.2%), lacerations (89; 14%), and motor vehicle injuries (78; 12.3%). In traumatic injuries and non-traumatic lesions, the ulnar nerve was the most commonly affected, followed by radial and median nerves. Direct causes of the nerve lesions were mainly compressions (289; 45.5%), elongation/irritation (214; 33.7%), and laceration (114; 18%), whereof 84 (13.2%) had full transections and 31 (4.9%) had incomplete transections, leaving the majority of 515 (81.1%) nerves intact.

At the level of the forearm including the wrist, 1925 nerve lesions were identified. Affected nerves were the median nerve (1169; 60.7%), ulnar nerve (481; 25%), radial nerve (253; 13.1%), and medial antebrachial cutaneous nerve (11; 0.6%). Concomitant injuries included 274 (14.2%) lacerations of one and 61 (5.2%) of two or more major vessels, 179 (9.3%) wounds with at least four damaged tendons followed by 100 (5.2%) cases with one, 78 (4.1%) with two, 57 (3%) with three, and 44 (2.3%) with four damaged tendons. Furthermore, 251 patients (13%) had fractures at the level of the forearm. Average inpatient treatment duration was 7.1 (15.2) days with a mean of 1.19 (0.89) operations. The main causes were lacerations (717; 37.2%), followed by non-traumatic injuries (669; 34.8%), falls (278; 14.4%), and 107 (5.6%) motor vehicle injuries. In traumatic and non-traumatic lesions, the median nerve followed by the ulnar and radial nerve were most commonly injured, but proportional distribution varied. Non-traumatic lesions affected the median nerve in 81.9%, whereas it was only 52.8% for the case in which traumatic injuries were followed by a higher proportion of traumatic ulnar nerve injuries (29.9%) vs. non-traumatic (16.2%).

A direct cause was identified in 1006 (52.3%) cases of compression, 604 (31.4%) cases of transections, which included 451 (23.4%) complete and 153 (7.9%) incomplete transections. In addition, 1316 (68.4%) nerves were found intact. Elongation/irritation was found in 233 (12.1%) cases.

At the level of the hand, a total of 2306 nerve lesions were found. Most common were lesions to finger nerves (2089; 90.6%), followed by the radial nerve (81; 3.5%), median nerve (70; 3%), and ulnar nerve (66; 2.9%). In addition, 91.7% of the lesions to finger nerves were caused by traumatic injuries. Hereby, 1457 (69.75%) single nerve injuries occurred, and 632 (30.25%) patients had multiple (two or more) affected finger nerves with the most common combination being N1 and N2 (74 cases, 3.5%) followed by N3 and N4 (74 cases, 3.5%) (N1 representing finger nerve 1, N2 finger nerve 2, etc.)

Concomitant injuries were found in the form of 925 (40.1%) lacerations of one and 179 (7.8%) of two and 230 (11%) of multiple (i.e., more than two) vessels. In addition, 972 (42.2%) nerve injuries had no accompanying vessel damage. We identified 508 (22%) wounds with one damaged tendon followed by 328 (14.2%) cases with two, 102 (4.4%) with three, and 120 (5.3%) with four or more damaged tendons.

Furthermore, 448 (19.4%) cases had fractures at the level of the hand. Average inpatient treatment duration was 5.1 (5.7) nights with a mean of 1.08 (0.5) operations.

**Figure 3:**
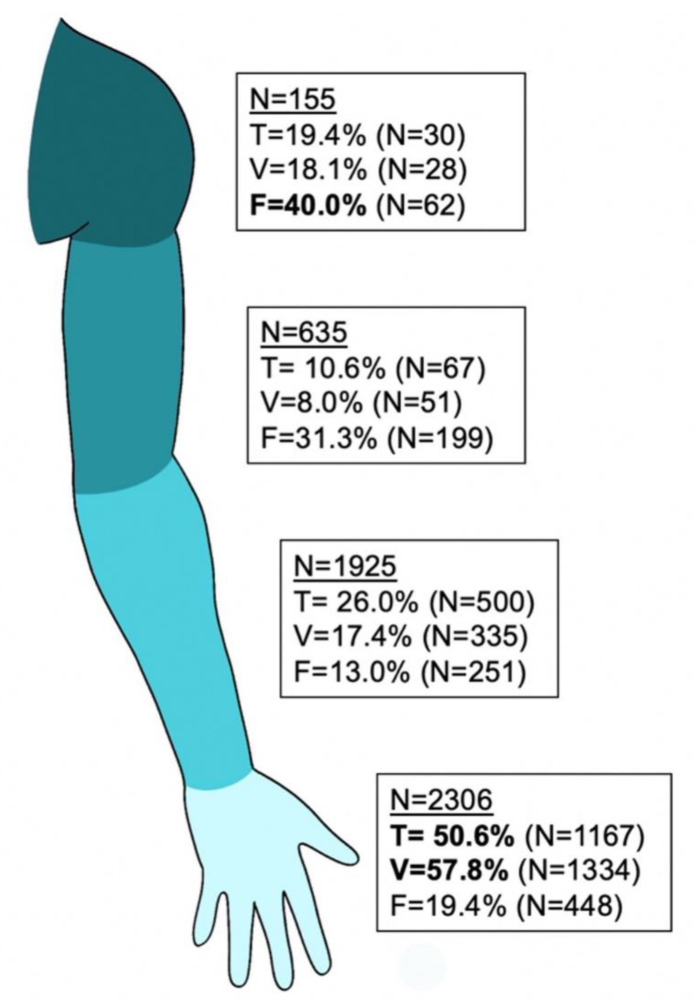
Concomitant injuries on the upper extremity. Displayed are injuries on different levels with concomitant tendon or muscle (T), blood vessel injuries (V) or fracture (F). The number of patients with at least one e.g., tendon injuries, is shown.

Main causes were lacerations (2105; 91.3%), followed by non-traumatic lesions (87; 3.8%), falls (27; 1.2%), and 24 (1%) motor vehicle injuries. Direct causes were identified in 2066 (89.6%) transections, of which 1843 (79.9%) were complete and 222 (9.6%) were incomplete transections, with 235 (10.2%) intact nerves. Elongation/irritation was found in 58 (2.5%) cases, neuroma formations in 71 (3.1%) and compressions in 99 (4.3%) cases (Figure 3).

#### 3.3.2. Lower Extremity

**Figure 4:**
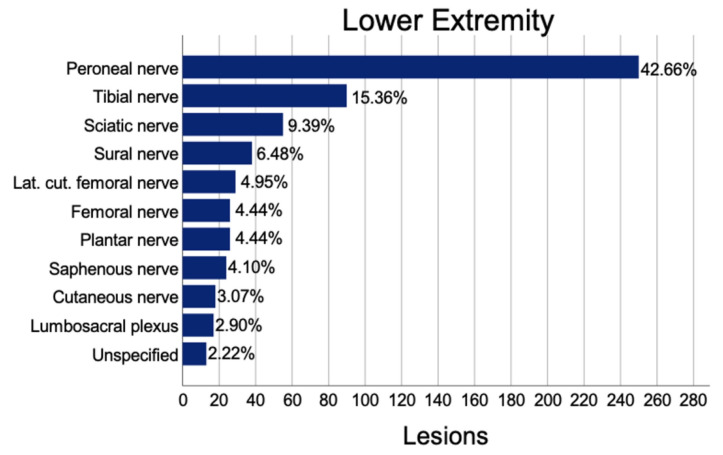
Prevalence of lesions to the lower extremity.

A total of 586 lower extremity nerve lesions were found. Thereof, we identified the peroneal nerve as the most commonly affected (250; 42.7%), followed by the tibial nerve (90; 15.4%), and the sciatic nerve (55; 9.4%). Twenty-two (3.8%) concomitant lacerations of one and 94 (12.3%) concomitant lacerations of two or more vessels were found. In addition, 492 (84%) lower extremity nerve injuries had no accompanying vessel damage. We identified 103 (17.6%) wounds with one or more damaged tendons. Furthermore, 204 (34.8%) cases were accompanied by a fracture. Average inpatient treatment duration was 18.2 (20.5) nights with a mean of 2.02 (2.29) operations (Figure 4).

The main causes were non-traumatic injuries (232; 39.6%), motor vehicle injuries (159; 27.1%), falls (59; 10.1%), lacerations (55; 9.4%), and neuroma formations (54; 9.2%). In addition, 275 (46.9%) elongation/irritations and 114 transections (19.5%), thereof 107 (18.3%) complete and 7 (1.2%) incomplete transections, were identified as direct causes. Furthermore, 447 (76.3%) were intact. Traumatic injuries mainly affected the peroneal nerve (48.9%) followed by tibial (16.4%) and sural nerve (8.5%), whereas non-traumatic lesions displayed 24.5% peroneal, 18.6% plantar nerve, and 15.7% tibial nerve affection.

Analyses of nerve lesions to the head and neck region revealed 81 cases. Thereof, 76 (93.8%) represented facial nerve lesions. All other injured nerves represented a variety of cutaneous nerves mostly damaged and treated in combination with traumatic lacerations. Average inpatient treatment was 14.7 (11.3) nights with a mean of 1.01 (1.12) operations.

### 3.4. Seasonal Distribution

**Figure 5:**
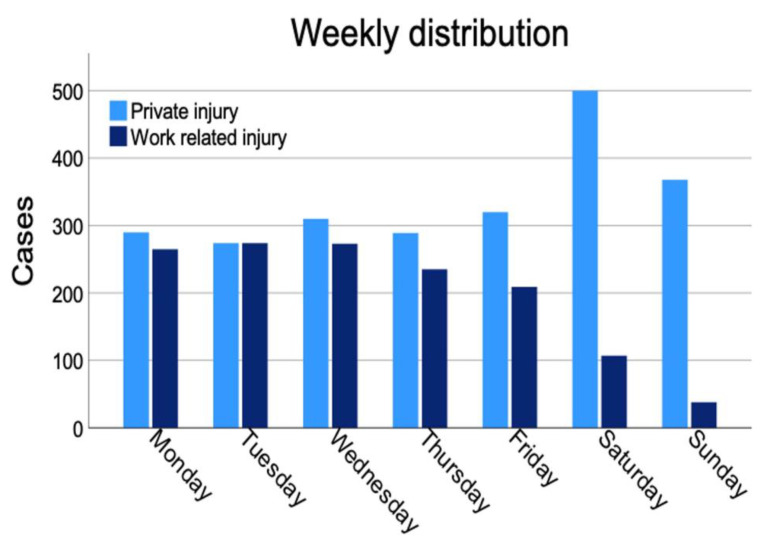
Timepoint of injury for work related and private injuries.

Analyses of the timepoints of the accident revealed that Saturday (21.3%) was the most likely day for a peripheral nerve injury occurring during a leisurely activity. (*p* < 0.001) (Figure 5).

**Figure 6:**
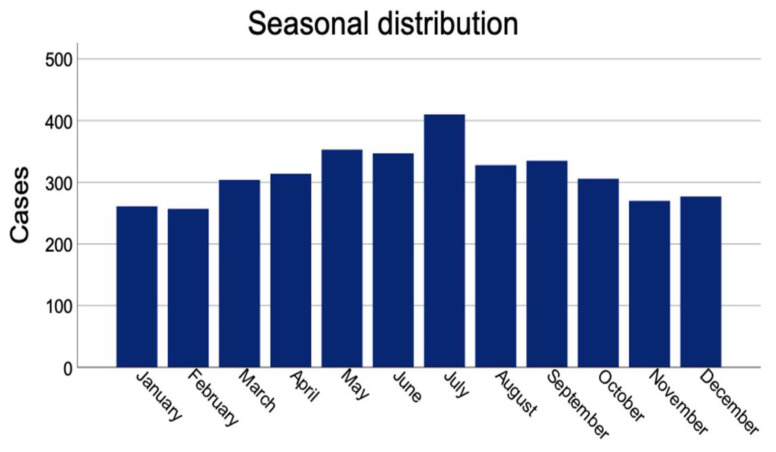
Seasonal distribution of all traumatic peripheral nerve injuries.

Work related injuries are more likely to happen from Monday to Wednesday and decline towards the weekend. A significant difference was found regarding the distribution of weekdays for private versus work-related injuries (*p* < 0.001). A yearly overview demonstrated a peak of peripheral nerve injuries in July and an overall trend towards higher injury incidences during summer months (Figure 6). A significant difference was seen in terms of months of trauma (*p* < 0.001).

### 3.5. Early Treatment

All traumatic injuries were evaluated whether they were treated within the first 24 h after trauma or not. Analyses showed that injuries at the level of the shoulder were in 90.8% not treated within 24 h after injury. Upper arm injuries were treated in 26.6% within 24 h. At forearm and wrist level 42.8%, and, at hand and finger level, 75.7% were treated within the first 24 h. Lower extremity injuries revealed in 15.8% an early treatment. Injuries to the head and neck region head had in 17.6% a treatment within 24 h after nerve injury. In total, 55.9% of all injuries were treated within 24 h.

## 4. Discussion

Nerve lesions are devastating conditions with a long regeneration process and often persisting impairment. This often results in long sick leave, occupational redeployment or, in case of work-related injuries, lifetime compensation. This causes not only high individual burden for the patient, but furthermore high socioeconomic costs for the society [1].

The exact epidemiology of peripheral nerve injuries remains unknown [3]. Current literature provides only partial information. Rosberg et al. describe hand injuries for a Swedish city either in general without peripheral nerve details [9] or with a circumscript patient number [7]. Other authors such as Kouyoumdjian et al. [10] provide a larger number of patients of 1124 cases in a 26 year time period but also with some limitations, as the large time period with significant evolvements in the healthcare system, treatment strategies and infrastructural development, and also with describing a south American population and infrastructure. As an example, he describes that 6.6% of the nerve injuries are a result of gunshot wounds, whereas we found only 0.5% of our nerve injuries being the result of gunshots and explosion combined. Other authors also describe epidemiological aspects of nerve injuries such as Birch et al. [11] but again with limited comparability of Afghan war injuries to central Europe. Other large collectives such as Huckhagel et al. [6] describe patient samples from nationwide registries but without further details on individual patients.

In order to overcome these gaps in literature and to create a representative picture of nerve lesion distribution and characteristics, we analyzed all 110,667 patients treated in our level I trauma center in central Europe. We found 5026 cases of nerve lesions which is, to our knowledge, the largest analysis of a trauma center worldwide.

We found in accordance with other studies significantly more young, male patients (67.7%) in the middle of their employment with lesions to the upper extremity (88.3%). In traumatic injuries, we even found more than 91% of injuries in the upper extremity. We previously published data on long-term costs of peripheral nerve injuries, indicating that about 30% of patients suffering from work related peripheral nerve injuries have more than 20% impairment in hand function requiring not only financial compensation but also potential redeployment. With the young age of the patients, this results in more than 30 years of compensation with an average cost of more than 100,000 Euro per patient during this period [1].

We found significantly more injuries to the left side (*p* < 0.001), as one would expect more left sided injuries due to more right-handed people (about 80–90% of the population) and the vast majority of injuries being digital nerve injuries caused by laceration and cuts [1,12].

We found a seasonal peak of injuries in July, which is slightly later than previously reported peaks from Rosberg et al. [13] who described their peak in May and June in a Scandinavian collective. They describe a peak of injuries on Saturday, which we only found in private injuries. Work related injuries peaked at the beginning of the week in our collective. Knowing of these seasonal and weekly peaks might influence the surgery planning in treating centers, but also enhance the necessity for prevention work and related education at the workplaces.

Considering the level of injury, more proximal injuries are more likely to be caused by severe trauma such as motor vehicle accidents compared to distal injuries being more prone to lacerations. This is supported by the higher number of fractures in proximal injuries. We found the radial nerve injured in 9.5% of traumatic injuries. In 3.2% of traumatic injuries, the radial nerve lesion was in the upper arm and accompanied in 62.1% of injuries with a humeral fracture.

Interestingly, we found more lesions of the ulnar nerve (54.6%) in the upper arm and elbow region than radial nerve (32.6%) as proposed by current literature [14]. Even with only traumatic injuries without compression syndromes of the elbow, we identified 50.4% as ulnar nerve and 33.6% as the radial nerve.

Considering nerve continuity in traumatic injuries of the upper arm, the median nerve was found intact in 52.2%, the ulnar nerve in 87.1% and the radial nerve in 74.8%. This is especially relevant for therapy planning and potential early exploration of nerves with higher probability of injury. Our findings show that the majority of nerve injuries at the level of shoulder and upper arm are in continuity, also indicating the necessity of a proper diagnostic tool. As most of these injuries are caused by severe trauma such as motor vehicle accidents or falling, concomitant injuries can mask nerve damage and therefore delay sufficient treatment. We found 74% of polytrauma patients with a nerve injury were injured in motor vehicle accidents. With the brachial plexus and peroneal nerve mostly affected, the mainly young male patients have a potential severe malfunction if the nerve injuries remain undiagnosed for too long. Hereby, new technologies such as improved MRI imaging could play a major role in diagnosis and evaluation of the regeneration process in the future [15]. As regeneration of the nerve at such a high injury level is usually incomplete, precise and early diagnostics can help in the decision-making process to offer, for example, distal nerve transfer strategies to the patient.

In the forearm, we found the median nerve in 64.9% of all traumatic injuries intact, whereas the ulnar nerve (49.2% intact) and the radial nerve (28.8% intact) had a higher chance of complete transection.

Thus, distal injuries have a significantly higher percentage of concomitant vessel and tendon injury. Hereby, only 42.2% of injuries to the finger nerves had no vessel damage, which also influences longer operation times and has potential influence on wound and nerve healing due to insufficient blood perfusion. Therefore, we strongly advocate intraoperative exploration and visualization of these structures especially in distal volar injuries. This might also explain the higher number of nerves treated within the first 24 h at our facility as in distal injuries’ nerve damage may be easier to diagnose. Proximal injuries had a very low percentage of treatment within 24 h after injury as these injuries are mainly caused by high velocity injuries where stabilization of the patient is more important and precise nerve diagnosis is often hampered by anesthesia or severe pain medication.

Some nerves were in general found with a higher number of complete transection than others. Hereby, the radial nerve (50.4%), the musculocutaneous nerve (68.8%) and digital nerves (83.8%) were often completely transected in traumatic injuries. For comparison, the median nerve was only found completely cut in 22.3%. In doubt, these nerves should be explored in detail and earlier.

Even for a large trauma center, we had almost 50% of nerve lesions being caused by other entities such as compression. Specialized centers should be familiar with treatment of such conditions as well as capable of potential salvage procedures such as tendon and nerve transfers for all entities of nerve lesion.

## 5. Limitations

The main limitation of this study is its retrospective character which depends on documentation during clinical routine. This also hampers consistent presentation of the outcome after injury. Large prospective collectives could improve that in the future with standardized data acquisition.

## 6. Conclusions

This work describes 5026 cases of peripheral nerve lesions in a central European collective. Hereby, predominately young, male patients with upper extremity injuries were affected, which explains the high medical and socioeconomic relevance of this topic. The data revealed a large number of concomitant injuries on an individual level of injury, which are at risk of being missed besides treatment of the overlying pathology but need very special attention. An early diagnosis and treatment are of utmost importance. Patients should be referred to a specialized center to enhance potential outcome and treatment. These centers have a higher capability of detailed diagnosis, early treatment and potential salvage treatment in case of insufficient regeneration.

## Figures and Tables

**Table 1:** Duration of inpatient treatment according to different nerve injuries. Trunk and lower extremity lesions show a longer duration of inpatient treatment. Nights spent in the hospital are shown. Therefore, a duration of two nights in hospital equals three days in hospital. This format was used due to internal regulations and to eliminate data irritation with outpatient treatment who have a day in the hospital but no nights.

	Nights in Hospital		
Nerv	Mean (d)	Standard Deviation	N
Lumbosacral plexus	36.41	20.38	17
Peroneal nerve	21.03	22.60	250
Sciatic nerve	20.78	12.14	55
Femoral nerve	20.62	17.27	26
Tibial nerve	18.37	21.83	90
Brachial plexus	17.94	19.13	128
Sural nerve	14.95	19.03	38
Facial nerve	14.61	11.52	76
Lat. cut. femoral nerve	13.31	18.78	29
Axillary nerve	11.12	10.23	17
Suprascapular nerve	11.00	6.89	5
Cutaneous nerve	10.45	18.86	20
Radial nerve	10.10	20.07	552
Musculocutaneous nerve	9.35	9.65	20
Ulnar nerve	7.92	16.03	839
Saphenous nerve	7.42	7.90	24
Median nerve	6.91	16.20	1255
Med. antebrachial cut. nerve	5.94	4.09	17
Finger nerves	4.96	5.63	2089
Plantar nerve	2.38	2.61	26

**Table 2:** Intraoperative findings demonstrate various percentages of nerve continuity according to affected nerve and level. Nerves where no intraoperative records could be obtained are not displayed in the table.

				Median Nerve	Ulnar nerve	Radial Nerve	Musculocutaneous Nerve	Brachialis Plexus	Finger Nerves	Medial Antebrachial Cutaneous Nerve	Axillary Nerve	Suprascapular Nerve	Thoracodorsal Nerve	Long thoracic Nerve	Cutaneous Nerve	Medial Brachial Cutaneous Nerve	Unspecified	Total
Shoulder	continuity	in continuity	N	1	2	6	1	94			7	2	1	1				115
			%	100	100	85.7	100	86.2			77.8	50.0	100	100				85.2
		incomplete transection	N	0	0	1	0	0			1	1	0	0				3
			%	0	0	14.3	0	0			11.1	25.0	0	0				2.2
		complete transection	N	0	0	0	0	15			1	1	0	0				17
			%	0	0	0	0	13.8			11.1	25.0	0	0				12.6
	Total		N	1	2	7	1	109			9	4	1	1				135
			%	100	100	100	100	100			100	100	100	100				100
Upper arm	continuity	in continuity	N	32	313	159	3	2		1	4				0	0	1	515
			%	57.1	90.2	78.3	33.3	100		16.7	100				0	0	100	81.7
		incomplete transection	N	7	16	8	0	0		0	0				0	0	0	31
			%	12.5	4.6	3.9	0	0		0	0				0	0	0	4.9
		complete transection	N	17	18	36	6	0		5	0				1	1	0	84
			%	30.4	5.2	17.7	66.7	0		83.3	0				100	100	0	13.3
	Total		N	56	347	203	9	2		6	4				1	1	1	630
			%	100	100	100	100	100		100	100				100	100	100	100
Forearm	continuity	in continuity	N	930	286	96	1			2					0		1	1316
			%	79.7	59.6	38.2	11.1			18.2					0		100	68.5
		incomplete transection	N	100	34	17	1			1					0		0	153
			%	8.6	7.1	6.8	11.1			9.1					0		0	8.0
		complete transection	N	137	160	138	7			8					1		0	451
			%	11.7	33.3	55.0	77.8			72.7					100		0	23.5
	Total		N	1167	480	251	9			11					1		1	1920
			%	100	100	100	100			100					100		100	100
Hand	continuity	in continuity	N	43	25	24			143									235
			%	61.4	37.9	29.6			6.9									10.2
		incomplete transection	N	9	6	1			206									222
			%	12.9	9.1	1.2			9.9									9.7
		complete transection	N	18	35	56			1734									1843
			%	25.7	53.0	69.1			83.2									80.1
	Total		N	70	66	81			2083									2300
			%	100	100	100			100									100

## Data Availability

Not applicable.
